# Activated microglia release β−galactosidase that promotes inflammatory neurodegeneration

**DOI:** 10.3389/fnagi.2023.1327756

**Published:** 2024-01-12

**Authors:** Emily J. A. Kitchener, Jacob M. Dundee, Guy C. Brown

**Affiliations:** Department of Biochemistry, University of Cambridge, Cambridge, United Kingdom

**Keywords:** microglia, β-galactosidase, neurodegeneration, senescence, neuroinflammation

## Abstract

Beta (β)-galactosidase is a lysosomal enzyme that removes terminal galactose residues from glycolipids and glycoproteins. It is upregulated in, and used as a marker for, senescent cells. Microglia are brain macrophages implicated in neurodegeneration, and can upregulate β-galactosidase when senescent. We find that inflammatory activation of microglia induced by lipopolysaccharide results in translocation of β-galactosidase to the cell surface and release into the medium. Similarly, microglia in aged mouse brains appear to have more β-galactosidase on their surface. Addition of β-galactosidase to neuronal-glial cultures causes microglial activation and neuronal loss mediated by microglia. Inhibition of β-galactosidase in neuronal-glial cultures reduces inflammation and neuronal loss induced by lipopolysaccharide. Thus, activated microglia release β-galactosidase that promotes microglial-mediated neurodegeneration which is prevented by inhibition of β-galactosidase.

## Introduction

The mammalian enzyme β-galactosidase hydrolyses terminal galactose residues from glycolipids and glycoproteins. In humans, β-galactosidase deficiency causes neurodegeneration and death during development due to accumulation of the glycolipid/ganglioside GM1, which is normally metabolized by β-galactosidase to the ganglioside GM2 (Bonten et al., 2014). Almost all cellular β-galactosidase is located within the lysosomes, where it forms a complex with neuraminidase-1 (Neu1) and Protective Protein/Cathepsin A (PPCA), which protects β-galactosidase from degradation within the lysosomes (Bonten et al., 2014). Neu1 hydrolyses terminal sialic residues from glycolipids and glycoproteins, to reveal terminal galactose residues, which are then hydrolyzed by β-galactosidase (Puigdellívol et al., 2020).

Senescent cells are known to have an increased β-galactosidase activity; the β-galactosidase activity of cells measured at pH 6.0 (known as “senescence-associated beta-galactosidase activity”) is commonly used as a marker of senescent cells, often detected by histochemical X-gal staining (Dimri et al., 1995). This was originally thought to be a β-galactosidase activity unique to senescent cells, but subsequently was shown to be due to the normal lysosomal β-galactosidase (i.e., the only β-galactosidase in mammals), which was found to be overexpressed in senescent cells, accounting for the increased X-gal staining of senescent cells (Lee et al., 2006).

The expression of lysosomes in cells (including expression of β-galactosidase) is regulated by transcription factor EB (TFEB), which also promotes the translocation of β-galactosidase to the plasma membrane/cell surface by lysosomal exocytosis (Magini et al., 2013). We recently reported that activation of microglia resulted in the translocation of Neu1 to the cell surface and extracellular space by lysosomal exocytosis (Allendorf and Brown, 2022). As Neu1 can be structurally and functionally coupled to β-galactosidase (Bonten et al., 2014), we were interested here in whether β-galactosidase was also released by activated microglia, and, if so, whether this had any functional consequences.

Microglia are resident brain macrophages and the main innate immune cell of the central nervous system. As such, microglia survey the brain for infection or damage, and if they detect these, become inflammatory activated in order to resolve the threat. However, chronic activation of microglia can be damaging to neurons and is implicated in many brain pathologies, including neurodegenerative diseases, such as Alzheimer’s disease and Parkinson’s disease (Thameem Dheen et al., 2007). Senescent microglia, overexpressing β-galactosidase, have been implicated in Alzheimer’s disease (Greenwood and Brown, 2021). And β-galactosidase associated with the neuronal membrane is increased in Alzheimer’s disease (Magini et al., 2015). β-galactosidase is increased in the CSF of Parkinson’s disease patients, indicating an increase in extracellular β-galactosidase in the brain (van Dijk et al., 2013). Extracellular and cell surface β-galactosidase can potentially degrade cell surface GM1, which is neuroprotective (van Dijk et al., 2013). GM1 also potently inhibits microglial activation (Galleguillos et al., 2022), and decreases in Parkinson’s disease (Chowdhury and Ledeen, 2022), potentially due to increased extracellular β-galactosidase in the PD brain (van Dijk et al., 2013).

In this study, we examined whether activated microglia release β-galactosidase, and, if so, whether β-galactosidase promoted inflammatory neurodegeneration.

## Materials and methods

### Cell culture and treatments

The immortalized cell line BV-2 (ECACC Cat# 0356, RRID:CVCL_0182) was maintained as previously described (Blasi et al., 1990; Shen et al., 2016). Neither cell line is listed as a commonly misidentified cell line by the International Cell Line Authentication Committee. Primary mixed neuron-glial cultures were prepared from the cerebellum of 3–5 day old Wistar rats (Charles River, RRID:RGD_2312511), following procedures described elsewhere (Carrillo-Jimenez et al., 2018; Allendorf et al., 2020). All animal experiments were approved by the Cambridge University Local Research Ethics Committee and undertaken in accordance with the UK Animals (Scientific Procedures) Act (1986).

Cells were treated as follows: adenosine triphosphate (ATP) was used at 1 mM and A23187 was used at 10 μM. LPS (100 ng/mL) and PMA (100 nM) were added over 18 or 72 h where indicated. Exogenous β-galactosidase from bovine liver (Sigma, St Louis, MO, USA) was used at 20 mU/mL for 72 h. For heat inactivation, β-galactosidase was first incubated at 70 °C for 5 min. For depletion experiments, mixed neuronal-glial co-cultures were treated 3 DIV with 5 μM PLX-3397 (PLX). β-galactosidase inhibitors, D-galactono-1,4-lactone (DGL, BioSynth, Staad, Switzerland) and 1-Deoxygalactonojirimycin (hydrochloride) (DGJ, Cambridge Bioscience) were used 1 mM or 10 mM for 72 h. Treatments were compared to a vehicle treated control group. The most appropriate vehicle was chosen for each experiment. Dimethyl sulfoxide (DMSO) for PLX treatments and phosphate buffered saline (PBS) for all other experiments.

### β-galactosidase activity assays

BV-2 microglia were seeded at 1 × 10^5^ cells/well in black, clear-bottom 96-well plates (Greiner) and treated as above. β-galactosidase activity on live cells was assessed in assay buffer adjusted to pH 7.0 or pH 4.0 for supernatant activity assays. The supernatant activity was assayed at final pH 4.7 as a result of diluting DMEM (which uses a sodium bicarbonate buffer system) with pH 4.0 assay buffer at a 1: 1 ratio. For the assay, media was removed from live cells and immediately replaced with 50 μL PBS. For supernatants, debris was removed by centrifugation 150 RCM, 5 min and 50 μL used for the assay. The assay was initiated by the addition of 50 μL of 2X assay buffer. Assay buffer (1X) contained 100 mM sodium phosphate (pH 7.0) or 100 mM citric acid/200 mM sodium phosphate buffer (pH 4.0), 1 mM MgCl2, 50 μM β-mercaptoethanol and 0.5 mg/mL 4-Methylumbelliferyl-β-D-galactopyranoside (MUG, GlycoSynth) in distilled water. 0.2 mU/mL β-galactosidase from bovine liver (Sigma, St Louis, MO, USA) was used as a positive control. Kinetic experiments were performed using a plate reader (FlexStation 3, Molecular Devices), pre-heated to 37°C, with fluorescence determinations beginning immediately upon the addition of assay buffer and taken every minute for 2 h, using 360/40 nm excitation and 460/40 nm emission filters. A standard curve was produced using 4-methylumbelliferone (MUB) which was used to convert arbitrary fluorescent units and calculate concentration of MUB produced in μM.

For assessing inhibitors of β-galactosidase, D-galactono-1,4-lactone and DGJ were diluted to 2X the indicated concentrations (1 mM or 10 mM) in PBS containing 2X the final concentration (0.2 mU/mL) of β-galactosidase from bovine liver, then the assay was initiated as above.

### Cell viability and density quantification

After treatment some culture media was removed and saved for analysis where necessary, then cell viability, defined as % necrotic (PI-positive) cells, was measured at indicated endpoints by differential dye uptake of propidium iodide (identifying necrotic cells) and Hoechst 33342 (identifying all cells) using a fluorescent microscope (EVOS M5000). Cell densities were quantified as described previously (Birkle and Brown, 2023). Briefly, Alexa Fluor-488 NeuO (Stemcell Technologies, Cambridge, UK) was used to identify neurons and Alexa Fluor-594 IB4 (Invitrogen, Paisley, UK) was used to identify microglia. Cultures were imaged using 10× objective, with four images taken in consistent positions around each well. Image sets were analyzed for the number of each cell type using a custom CellProfiler (Stirling et al., 2021b)/CellProfiler Analyst (Stirling et al., 2021a) pipeline.

### BV-2 cell viability and density quantification

BV-2 microglia were seeded at 5 × 10^3^ in 96-well plates and treated with vehicle or 20 mU/mL β-galactosidase from bovine liver (Sigma, St Louis, MO, USA) for 48 h. Density and cell viability, defined as % necrotic (PI-positive) cells, was measured at 0, 24, and 48 h by differential dye uptake of propidium iodide (identifying necrotic cells) and Hoechst 33342 (identifying all cells) using a fluorescent microscope (EVOS M5000). Cultures were imaged using a 10× objective, with four images taken in consistent positions around each well. Images were analyzed using FIJI.

### Measurement of β-galactosidase and inflammatory cytokines

Following treatments, β-galactosidase concentration from BV-2 supernatants and β-galactosidase concentration from mixed neuronal-glial cultures was determined using Mouse Beta-Galactosidase (GLB1) ELISA Kit (Abbexa, Cambridge, UK) and Rat Beta-Galactosidase (GLB1) ELISA Kit (Abbexa, Cambridge, UK), respectively, according to the manufacturer’s instructions. TNFα and IL-6 detection was achieved by ELISA as per the manufacturer’s instructions (BioLegend, San Diego, CA, USA).

### Immunohistochemistry and imaging of free-floating brain slices

Transcardial perfusion, tissue sectioning, tissue staining, image acquisition and analysis were done as previously described (Dundee et al., 2023). Briefly, 25 μm serial coronal sections were prepared using a sliding microtome and stored in PBS containing 0.025% sodium azide as free-floating sections. For each of five wild-type (WT) mice, aged 4 and 17 months old, three sections were used, taken 300 μm apart per mouse. Sections were incubated with mouse anti-Iba1 (1:200, Sigma, St Louis, MO, USA), rabbit anti-β-galactosidase (1:200, Thermo Fisher, Waltham, MA, USA) and rat anti-CD68 (1:200, Thermo Fisher, Waltham, MA, USA) antibodies in blocking solution for 2 h at 37°C (Xiao et al., 2017), then washed with PBS and incubated for 2 h at 37°C with secondary antibodies, Alexa Fluor-488 goat anti-mouse (1:200, Thermo Fisher, Waltham, MA, USA), Alexa Fluor-568 goat anti-rabbit (1:200, Thermo Fisher, Waltham, MA, USA) and Alexa Fluor-647 goat anti-rat (1:200, Thermo Fisher, Waltham, MA, USA). Sections were washed with PBS and mounted on poly-L-lysine treated glass slides in DAPI-containing Vectashield mounting medium (Vector Laboratories, Newark, CA, USA) and imaged on a confocal microscope (Nikon C2si, 63×, 1.35 NA oil immersion objective using 405, 488, and 561 nm lasers). For imaging, Z-stacks (0.5 μm step intervals) were taken of the somatosensory cortex. Fifteen microglia were analyzed across the three sections from each mouse. Image analysis was done in FIJI and Imaris (version 9.1.2). Briefly, background subtraction and intensity normalization were done, then microglial surface rendering (using Iba1 staining) and β-galactosidase staining intensity within Iba1+ structures was analyzed in Imaris. The results for the surface-rendered objects were represented as volume (μm^3^) and β-galactosidase intensity as MFI.

### Statistical analysis

Statistical analysis was performed using GraphPad Prism (version 9.0) and data was collected from a minimum of 3 independent experiments. Shapiro–Wilk test of normality was performed. Statistical significance was assessed by *t*-test, or repeated measures one-way or two-way ANOVA, followed by Šídák’s or Dunnett’s *post-hoc* test (see figure legends). Error bars represent the standard error of the mean of experiments (SEM). *p*-values refer to the probability of the null hypothesis that the means do not differ. *p* < 0.05 was considered significant, and *p* ≥ 0.05 not significant.

## Results

### Activation of microglia induces β-galactosidase release

**FIGURE 1:**
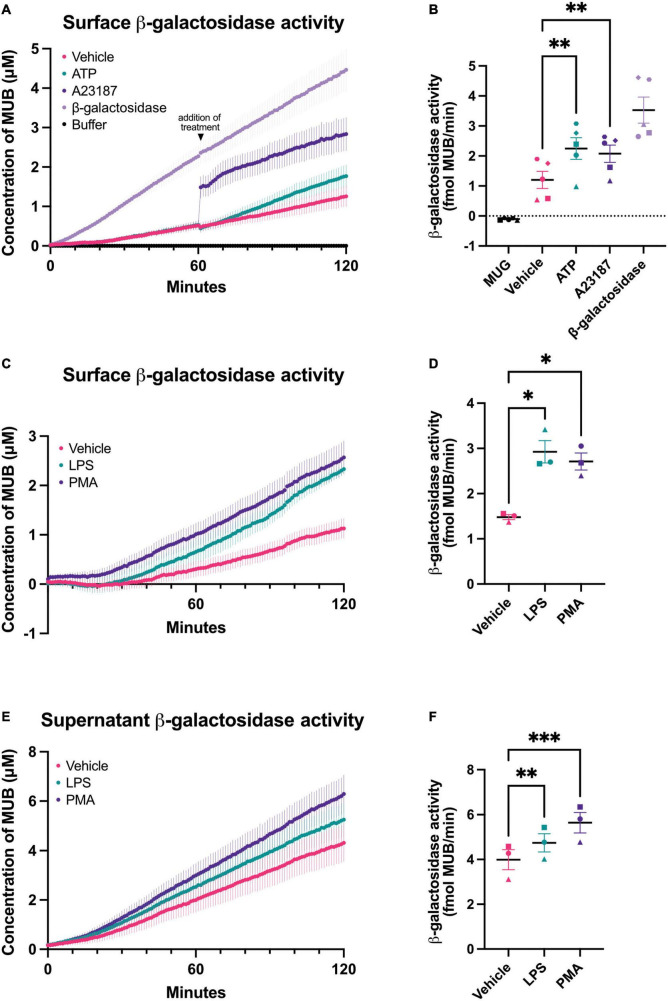
Chronic and acute activation of BV-2 microglia induces β-galactosidase release. β-galactosidase activity on the surface of BV-2 cells and released into the supernatant was determined using the rate of MUG to MUB conversion when added to a monolayer of cells or to the cleared supernatant from these cells. β-galactosidase activity on live cells was assessed in assay buffer adjusted to pH 7.0 or pH 4.0 for supernatant activity assays. Buffer refers to the 1X assay buffer containing 0.5 mg/mL MUG in the absence of cells (negative control). β-galactosidase (0.2 mU/mL) from bovine liver was used as a positive control for the assay. **(A)** Cells were acutely treated with ATP (1 mM) or A23187 (10 μM) 1 h after the initiation of the MUG assay and the rate of MUG-MUB conversion after treatment addition was calculated in **(B)**. **(C)** BV-2 cells were treated with LPS (100 ng/mL) or PMA (100 nM) for 18 h, then supernatant was removed and β-galactosidase activity was assayed on the cell surface. **(D)** The rate at which MUG was converted to MUB was determined from **(C)**. **(E)** The supernatants of LPS and PMA treated cells from **(C)** were cleared and assayed for β-galactosidase activity and rate determined in **(F)**. Data represents mean values ± SEM of at least 3 independent experiments. Statistical comparisons were made by repeated measures one-way ANOVA with Dunnett’s *post hoc* comparisons test. Asterisk (*) indicate significance compared to untreated control (*p < 0.05, **p < 0.01, ***p < 0.001).

It is unknown whether intact microglia can release β-galactosidase, so we measured this with BV-2 microglia and primary microglia in various conditions. To measure cell surface β-galactosidase activity, we added the β-galactosidase substrate, 4-methylumbelliferyl-β-D-galactopyranoside (MUG), to a monolayer of live BV-2 cells in the well of their cell culture plate, and followed the rate at which MUG was converted to the fluorogenic product 4-methylumbelliferone (MUB) in a plate reader. MUG is cell impermeant, so when added to intact cells, the assay only measures the cell surface or extracellular activity (Aureli et al., 2009). The culture medium was changed to PBS just prior to the assay, so that only cell surface or acute release of β-galactosidase was measured by the assay. In the absence of cells, there was no measurable β-galactosidase activity, but in the presence of live BV-2 cells, there was a significant β-galactosidase activity, which very slowly increased with assay time (Figure 1A). This indicates that there is a β-galactosidase activity present on the surface of BV-2 microglia. Addition of β-galactosidase isolated from bovine liver to the assay in the absence of cells resulted in steady activity (Figures 1A, B). The cell surface β-galactosidase activity was measured at pH 7.0, and therefore is likely to be active extracellularly.

One potential cause of β-galactosidase release from lysosomes is lysosomal exocytosis (Magini et al., 2013), which can be induced by a rise in intracellular calcium (Reddy et al., 2001). To induce a rise in intracellular calcium, we added A23187 (a calcium ionophore) or ATP (a P2X7 agonist) to BV-2 microglia. Addition of A23187 induced an immediate, artifactual increase in fluorescence due to the fluorescence of the compound itself, then an increase in rate for 5–10 min, and finally a return to baseline rate. ATP induced an increase in rate for about 60 min (Figure 1B). This is consistent with an intracellular calcium rise causing β-galactosidase release by lysosomal exocytosis.

To test whether β-galactosidase is released from inflammatory activated microglia, we treated BV-2 microglia for 18 h with lipopolysaccharide (LPS). LPS induced a significant increase in β-galactosidase activity on the surface of the cells, compared to cells not treated with LPS (Figures 1C, D). We also measured the β-galactosidase activity released from the cells into the cell culture medium during the course of the 18 h incubation ± LPS, and we found that LPS induced a significant increase in extracellular β-galactosidase activity (Figures 1E, F), consistent with LPS-induced release from microglia.

Phorbol 12-myristate 13-acetate (PMA) is a protein kinase C activator that has been reported to induce β-galactosidase and senescence of microglia (Cao et al., 2020). So, we tested whether PMA treatment of microglia could induce β-galactosidase release, and found that BV-2 microglia cultured with PMA had significantly more β-galactosidase activity on the cell surface (Figures 1C, D) and released significantly more β-galactosidase activity into the culture medium (Figures 1E, F).

**FIGURE 2:**
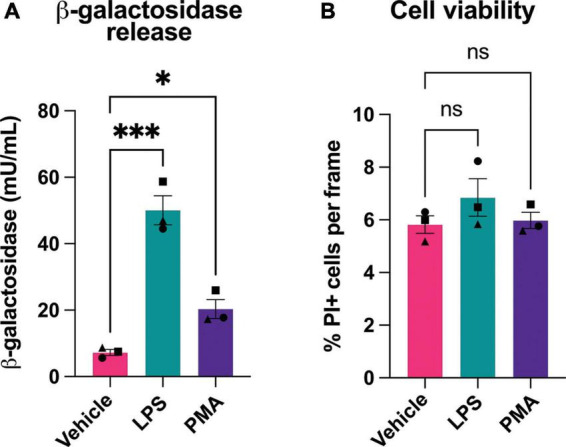
Microglial activation induces release of β-galactosidase protein without affecting cell death. **(A)** BV-2 cells were treated with LPS (100 ng/mL) or PMA (100 nM) for 18 h, then supernatant was collected and β-galactosidase concentration was determined by ELISA. **(B)** Cell viability of BV-2 microglia, post-treatment ± LPS or PMA, was assessed by propidium iodide uptake and presented as % of total cells (determined by Hoechst staining). Data represents mean values ± SEM of 3 independent experiments. Statistical comparisons were made by repeated measures one-way ANOVA with Dunnett’s *post hoc* comparisons test. Asterisk (*) indicate significance compared to untreated control (ns: *p* ≥ 0.05, *p < 0.05, ***p < 0.001).

The above assay measured β-galactosidase activity, but not protein. To measure whether β-galactosidase protein was released from activated microglia, we assayed β-galactosidase protein by ELISA in the cell culture media of BV-2 microglia pretreated with LPS or PMA. We found that PMA increased extracellular β-galactosidase protein 3 fold, and LPS increased extracellular β-galactosidase protein 7 fold, as measured by ELISA (Figure 2A). As cell death might, in principle, release β-galactosidase from lysosomes into the medium, we also checked whether LPS or PMA induced cell death, but there was no such increase in cell death (Figure 2B). Thus, activated microglia can release β-galactosidase to their surface and into their extracellular environment.

As BV-2 microglia release β-galactosidase into their extracellular medium, we tested the effect of adding isolated β-galactosidase to the extracellular medium of BV-2 microglia. We added 20 mU/mL β-galactosidase (isolated from bovine liver) to proliferating cultures of BV-2 cells, and measured cell density and cell death over 48 h. β-galactosidase had no significant effect on cell density or the increase in cell density caused by proliferation, and induced no cell death, as measured by propidium iodide staining of the cells (Supplementary Figure 1).

**FIGURE 3:**
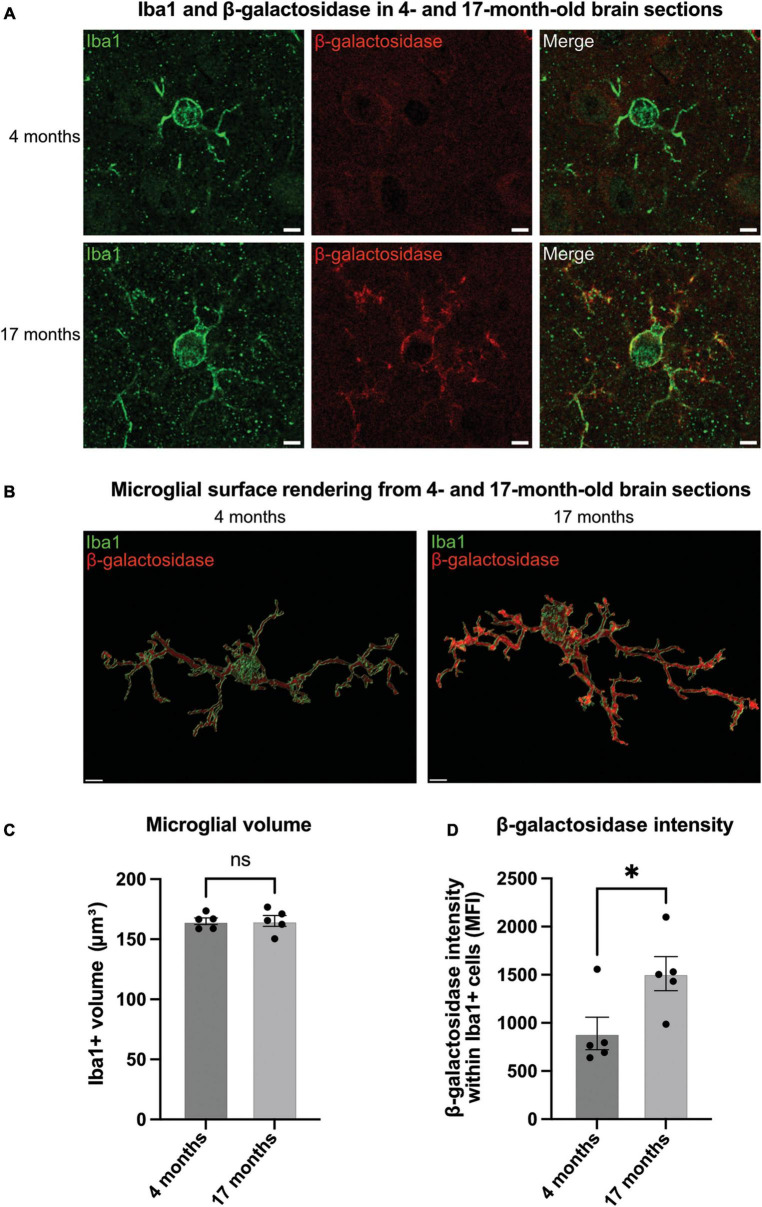
Microglia in aged brains have increased levels of β-galactosidase. **(A)** Representative confocal microscopy images of microglia from 4- to 17-month-old mice stained for Iba1 (green, microglial marker) and β-galactosidase (red) in the somatosensory cortex. Scale bar = 2 μm. **(B)** Representative renders of microglia from confocal images generated in Imaris. Scale bar = 2 μm. **(C)** Microglial volume as measured by area (μm^3^) of Iba1 staining. **(D)** Mean fluorescence intensity (MFI) of β-galactosidase within Iba1-positive microglia. Each point represents one animal comprised of 15 microglia analyzed across three equidistant sections. Error bars represent SEM and statistical comparisons were made via unpaired *t*-test. Asterisk (*) indicate significance (ns: *p* ≥ 0.05, *p < 0.05).

Having found that microglial activation can induce β-galactosidase on the microglial cell surface in culture, we wanted to test this *in vivo*. Microglia are known to become activated and senesce with age in mice (Matsudaira et al., 2023). So, we examined sections from the brains of mice at 4- and 17-months-old. For this, coronal brain slices were immunostained using antibodies to β-galactosidase and Iba1 (microglial marker) (Figures 3A, B). There was no significant difference with age in microglial Iba1 volume, indicating no effect of age on microglial size (Figure 3C). We then analyzed the staining intensity of β-galactosidase in Iba1-positive cells in the somatosensory cortex using confocal microscopy. We found that the intensity of β-galactosidase on Iba1-positive structures was significantly increased in 17-month-old compared with 4-month-old mice (Figure 3D), indicating a significant increase in β-galactosidase levels with age. Much of the β-galactosidase staining appeared to be on the microglial surface in aged brains, rather than in intracellular lysosomes (Figure 3A), consistent with translocation to the cell surface. This was further supported by staining of slices with Iba1 and the lysosomal marker, CD68 (Supplementary Figure 2). This confirmed that although lysosomes appear larger in aged brains, the lysosomes are still largely confined to the perinuclear cell body (Supplementary Figure 2), and their distribution is distinct from that of β-galactosidase in aged brains (Figure 3).

### Primary cultures release β-galactosidase, and added β-galactosidase promotes neuronal loss and microglial activation

**FIGURE 4:**
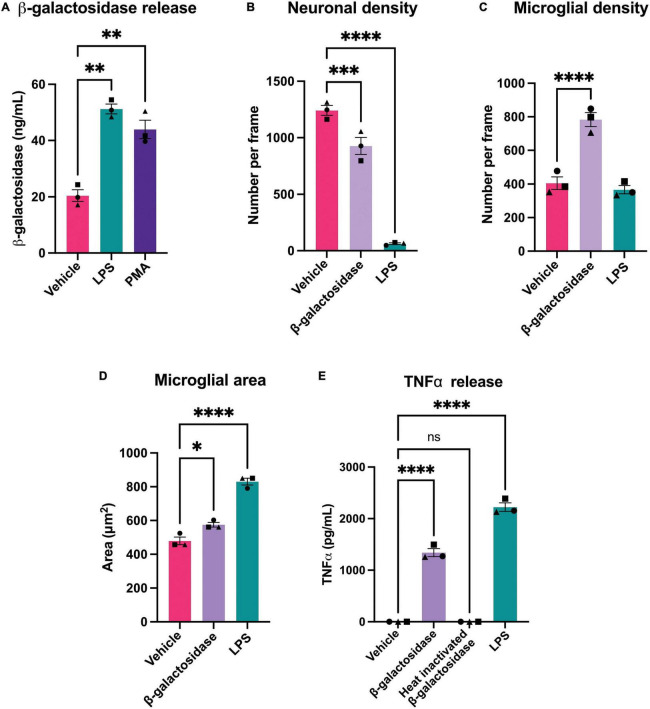
β-galactosidase promotes neuronal loss and microglial activation. **(A)** Mixed neuronal-glial cultures were treated with LPS (100 ng/mL) or PMA (100 nM) for 72 h and supernatants were assessed for β-galactosidase levels by ELISA. **(B)** Mixed neuronal-glial cultures were treated with β-galactosidase (20 mU/mL) and LPS (100 ng/mL) for 72 h then cultures were stained with Hoechst 33342 (to identify nuclei and apoptotic cells), isolectin B4 (to identify microglia), NeuO (to identify live neurons) and live neuronal cell numbers quantified using CellProfiler. **(C)** Microglial cell numbers quantified by fluorescent microscopy using CellProfiler. **(D)** Microglial cell area was quantified in CellProfiler. **(E)** Mixed neuronal-glial cultures were treated with β-galactosidase (20 mU/mL), heat inactivated β-galactosidase (20 mU/mL) and LPS (100 ng/mL) for 72 h then supernatants were assessed for TNFα levels by ELISA. Data represents mean values ± SEM of 3 independent experiments. Statistical comparisons were made by repeated measures one-way ANOVA with Dunnett’s *post hoc* comparisons test. Asterisk (*) indicate significance compared to untreated control (ns: *p* ≥ 0.05, *p < 0.05, **p < 0.01, ***p < 0.001, ****p < 0.0001).

As activated microglia released β-galactosidase, we were interested in whether this released β-galactosidase could contribute to neurodegeneration in primary neuronal-glial cultures. These cultures were isolated from the cerebellum of P3-P5 rats and cultured for 7 days (Carrillo-Jimenez et al., 2018), and contain 83 ± 3% neurons, 11 ± 2% astrocytes and 4 ± 1% microglia (Kinsner et al., 2005). We first measured whether LPS or PMA would induce β-galactosidase release in these cultures. We found that addition of 100 ng/mL LPS or 100 nM PMA induced a significant increase in β-galactosidase protein in the culture medium (Figure 4A).

As LPS and PMA induced an increase in extracellular β-galactosidase, we next tested whether addition of isolated β-galactosidase affected neuronal viability and microglial activation in mixed neuronal-glial cultures. We added 20 mU/mL β-galactosidase (or 100 ng/mL LPS for comparison) to these cultures for 72 h. Addition of β-galactosidase induced a loss of about 25% of the neurons over 72 h (Figure 4B and Supplementary Table 1), and doubled the number of microglia in the cultures, compared to the untreated control (Figure 4C and Supplementary Table 1). β-galactosidase also changed microglial area and morphology in a similar way (but lower extent) to LPS (Figure 4D and Supplementary Figure 3).

Having found that microglia proliferate and undergo morphological changes with β-galactosidase addition, both indicators of activation, we tested whether β-galactosidase can induce release of pro-inflammatory cytokines by measuring the amount of extracellular TNFα (tumor necrosis factor alpha) by ELISA. Untreated cultures had no detectable TNFα; LPS-treated cultures had a high level of TNFα; while cultures treated with 20 mU/mL β-galactosidase had an intermediate level of TNFα (1342 pg/mL) (Figure 4E). To test whether the effects of added β-galactosidase were due to the β-galactosidase activity, rather than the protein or potential contaminants, we added heat-inactivated β-galactosidase, and found this had no effect on TNFα release (Figure 4E), neuronal or microglial count, or microglial morphology (Supplementary Figure 4). Hence, exogenous β-galactosidase activity promotes neuronal loss and activates microglia to a pro-inflammatory state.

**FIGURE 5:**
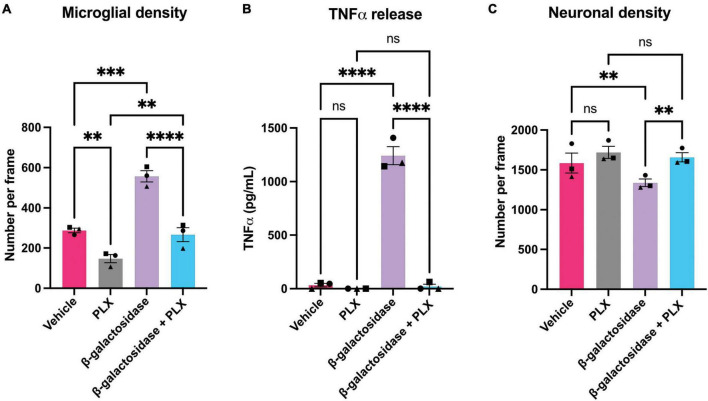
Microglial depletion prevents β-galactosidase-induced TNFα release and neuronal loss. Mixed neuronal-glial cultures were treated with PLX-3397 (5 μM) 3 DIV, then ± β-galactosidase (20 mU/mL) 7 DIV for 72 h. Cultures were then stained with Hoechst 33342 (to identify nuclei and apoptotic cells), isolectin B4 (to identify microglia), NeuO (to identify live neurons). **(A)** Microglial cell numbers were quantified by fluorescent microscopy using CellProfiler. **(B)** Supernatants were assessed for TNFα levels by ELISA. **(C)** Live neuronal cell numbers were quantified by fluorescent microscopy using CellProfiler. Data represents mean values ± SEM of 3 independent experiments. Statistical comparisons were made by repeated measures two-way ANOVA with Šídák’s *post hoc* comparisons test. Asterisk (*) indicate significance compared to untreated control (ns: *p* ≥ 0.05, **p < 0.01, ***p < 0.001, ****p < 0.0001).

To investigate whether the effects of β-galactosidase on neurotoxicity and inflammation require microglia, we depleted microglia in the mixed neuronal-glial cultures using PLX-3394, a colony stimulating factor 1 receptor (CSF1R) inhibitor, prior to β-galactosidase treatment. PLX treatment depleted the microglia in these cultures by at least 50% both in the absence and presence of exogenous β-galactosidase treatment, as measured using fluorescent isolectin-B4 to specifically label and quantify microglia (Figure 5A and Supplementary Table 2). And PLX treatment completely prevented the dramatic increase in TNFα levels induced by addition of β-galactosidase (Figure 5B). Furthermore, depletion of microglia prevented the loss of neurons induced by addition of β-galactosidase (Figure 5C and Supplementary Table 2). Together, these results suggest β-galactosidase is not directly neurotoxic, but that β-galactosidase induced neuronal loss is mediated by the activation of microglia and subsequent inflammation.

### Inhibiting β-galactosidase protects against LPS-induced neuronal loss and microglial activation

**FIGURE 6:**
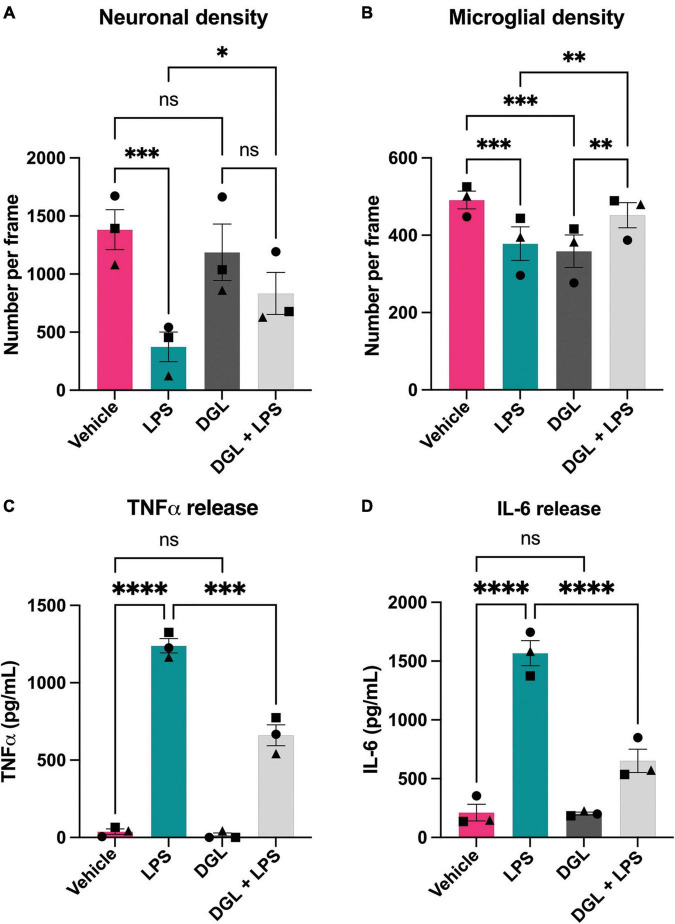
D-galactono-1,4-lactone protects against neuronal loss and microglial activation. Mixed neuronal-glial cultures were treated with LPS (100 ng/mL) ± 10 mM D-galactono-1,4-lactone (DGL) for 72 h then cultures were stained with Hoechst 33342 (to identify nuclei and apoptotic cells), isolectin B4 (to identify microglia), NeuO (to identify live neurons). **(A)** Live neuronal cell numbers and, **(B)** microglial cell numbers were quantified by fluorescent microscopy using CellProfiler. Supernatants were assessed for TNFα **(C)** and IL-6 **(D)** levels by ELISA. Data represents mean values ± SEM of 3 independent experiments. Statistical comparisons were made by repeated measures two-way ANOVA with Šídák’s *post hoc* comparisons test. Asterisk (*) indicate significance compared to untreated control (ns: *p* ≥ 0.05, *p < 0.05, **p < 0.01, ***p < 0.001, ****p < 0.0001).

As LPS induced β-galactosidase release in primary cultures, and added β-galactosidase induced microglial activation and neuronal loss, we wanted to test whether inhibiting β-galactosidase activity would affect LPS-induced microglial activation and neuronal loss. To do this, we tested the ability of several commercially available β-galactosidase inhibitors to inhibit the activity of isolated bovine β-galactosidase, and found two structurally dissimilar inhibitors, D-galactono-1,4-lactone (DGL) and 1-deoxygalactonojirimycin (hydrochloride) (DGJ), which significantly reduced activity of exogenous β-galactosidase (Supplementary Figure 5). We then tested whether these inhibitors affected the neuronal loss induced by LPS. Addition of LPS to mixed neuronal-glial cultures for 72 h resulted in neuronal loss as expected, but co-treatment with 10 mM D-galactono-1,4-lactone significantly protected against this loss (Figures 6A, B). D-galactono-1,4-lactone also reduced the effects of LPS on: TNFα levels (Figure 6C) and IL-6 levels (Figure 6D). Thus, inhibition of β-galactosidase by D-galactono-1,4-lactone reduced LPS-induced neuronal loss and microglial activation.

**FIGURE 7:**
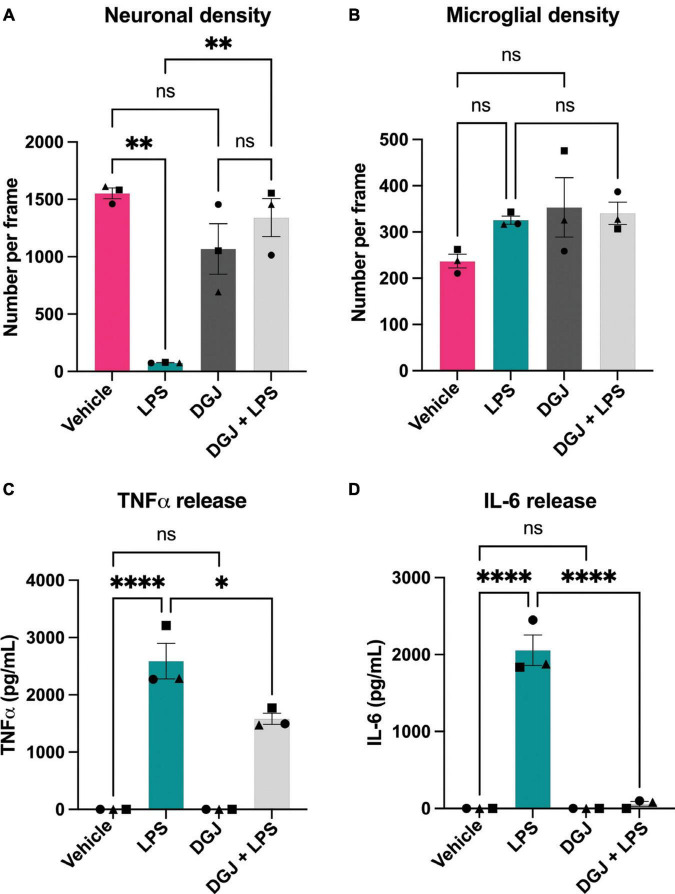
1-Deoxygalactonojirimycin protects against neuronal loss and microglial activation. Mixed neuronal-glial cultures were treated with LPS (100 ng/mL) ± 10 mM 1-Deoxygalactonojirimycin (DGJ) for 72 h then cultures were stained with Hoechst 33342 (to identify nuclei and apoptotic cells), isolectin B4 (to identify microglia), NeuO (to identify live neurons). **(A)** Live neuronal cell numbers and, **(B)** microglial cell numbers were quantified by fluorescent microscopy using CellProfiler. Supernatants were assessed for TNFα **(C)** and IL-6 **(D)** levels by ELISA. Data represents mean values ± SEM of 3 independent experiments. Statistical comparisons were made by repeated measures two-way ANOVA with Šídák’s *post hoc* comparisons test. Asterisk (*) indicate significance compared to untreated control (ns: *p* ≥ 0.05, *p < 0.05, **p < 0.01, ****p < 0.0001).

Similarly, we assessed the effects of the β-galactosidase inhibitor DGJ on mixed neuronal-glial cultures. Again, addition of LPS for 72 h resulted in neuronal loss, and co-treatment with 10 mM DGJ substantially protected against this loss (Figure 7A). DGJ caused no significant change in microglial numbers (Figure 7B), but reduced LPS-induced release of TNFα (Figure 7C) and LPS-induced release of IL-6 (Figure 7D).

## Discussion

It has previously been shown that LPS induces β-galactosidase protein and activity in BV-2 microglia (Yu et al., 2012; Borgonetti and Galeotti, 2022). We found that inflammatory activation of BV-2 microglia with LPS induced release of β-galactosidase onto the cell surface of microglia and into the medium, as measured by β-galactosidase protein or β-galactosidase activity. LPS also induced β-galactosidase release in primary neuronal-glial cultures, and addition of isolated β-galactosidase to these cultures induced microglial activation and neuronal loss, which was prevented by depleting microglia from these cultures. LPS-induced microglial activation and neuronal loss in these primary cultures was substantially reduced by two different β-galactosidase inhibitors. Overall, this suggests that LPS-activated microglia release β-galactosidase, which promotes microglial toxicity to neurons.

We did not investigate the mechanism by which LPS induces β-galactosidase release, but it has previously been shown that β-galactosidase can be released by lysosomal exocytosis (Magini et al., 2013), and that LPS can induce lysosomal exocytosis by microglia (Liu et al., 2008; Allendorf and Brown, 2022). Consistent with this, A23187 and ATP, which can induce a calcium rise and lysosomal exocytosis (Jaiswal et al., 2002), caused an acute increase in cell surface β-galactosidase activity. However, confirming that release was by lysosomal exocytosis would require further study.

Addition of β-galactosidase to primary cultures caused: increased microglia, altered microglial morphology, release of TNFα, and loss of neurons. We do not know why β-galactosidase increased microglial numbers, but microglial proliferation is commonly associated with microglial activation, and the TNFα release induced by addition of β-galactosidase is a potent mitogen for microglia (Mander et al., 2006). Hence, β-galactosidase might indirectly promote microglial proliferation by inducing microglial activation. However, a proliferative effect of β-galactosidase would need to be confirmed using a proliferation marker, such as Ki67. β-galactosidase altered microglial morphology as indicated by a flattening down of the microglia onto the culture well, but it might be useful to further characterize this morphological transition, for example by Scholl analysis.

Depletion of microglia from the primary cultures using PLX, prevented β-galactosidase-induced release of TNFα and loss of neurons. PLX is known to specifically deplete microglia without affecting astrocytes (Van Zeller et al., 2022), and we confirmed a depletion of microglia with no significant effect on neurons (Figure 5C) or astrocytes (Supplementary Figure 6). Therefore, β-galactosidase-induced neuroinflammation and neuronal loss requires microglia. This suggests that β-galactosidase activated microglia in a way that promoted neuronal loss. We did not further investigate the mechanism of this microglial activation or neuronal loss. However, mammalian β-galactosidase functions to remove terminal galactose residues from glycolipids and glycoproteins, including particularly the ganglioside GM1 (Li and Li, 1999). GM1 is anti-inflammatory in microglia and neuroprotective, both in culture and *in vivo* (van Dijk et al., 2013; Galleguillos et al., 2022). Thus, it is possible that cell surface β-galactosidase degrades GM1, resulting in microglial activation and neuronal loss. However, testing this would require further investigation, for example by staining for GM1 on microglia, and extracellular β-galactosidase may remove terminal galactose residues from receptors and other cell surface glycoproteins to change microglial and neuronal function. The mechanism of neuronal loss induced by β-galactosidase in these cultures was not further investigated, but possibilities include: (i) release of TNFα and IL-6, which are both sufficient to induce neuronal loss (Conroy et al., 2004; De Lella Ezcurra et al., 2010; Smith et al., 2012; Neniskyte et al., 2014), (ii) microglial phagocytosis of the neurons, induced by microglial activation (Neher et al., 2011), and (iii) exposure of N-acetylglucosamine residues on the cell surface, which may induce phagocytosis or complement activation (Cockram et al., 2021).

Lipopolysaccharide (LPS) induced β-galactosidase release in primary mixed neuronal-glial cultures, and two different β-galactosidase inhibitors reduced LPS-induced neuronal loss in these cultures. This suggests that β-galactosidase, in particular extracellular β-galactosidase, may be a good target to prevent inflammatory neurodegeneration. Unfortunately, these two inhibitors, D-galactono-1,4-lactone and DGJ, had to be used at millimolar levels, as this was the concentration at which they inhibited isolated β-galactosidase activity. We found no other commercially-available inhibitors of mammalian β-galactosidase that were more potent. Thus, further testing and translating the role of β-galactosidase may require the development of more potent inhibitors or other methods. Targeting all β-galactosidase may be toxic long-term, because lysosomal β-galactosidase is beneficial by degrading glycolipids and glycoproteins (Bonten et al., 2014), and lysosomes may be upregulated in microglia in a variety of conditions, for example to degrade cellular debris or amyloid beta. However, it might in principle be possible to specifically target extracellular β-galactosidase, using drugs that do not enter cells, or to target inhibitors to specific cell types, using for example myeloid-specific nanosystems (Zhu et al., 2022). For example, cell-impermeant β-galactosidase inhibitors could be injected into the brain with LPS to test whether inhibition of extracellular β-galactosidase prevents LPS-induced neuronal loss in rodents (Neher et al., 2014).

Senescent cells are characterized by the overexpression of β-galactosidase and lysosomes generally (Dimri et al., 1995; Kurz et al., 2000). This can be driven by the transcription factor TFEB, which can also promote lysosomal exocytosis (Magini et al., 2013). Thus, it is possible that part of the β-galactosidase in senescent cells could end up on the cell surface. This is at least consistent with our findings that: (i) PMA and LPS induce β-galactosidase release [as both PMA and LPS are reported to induce microglial senescence (Yu et al., 2012; Cao et al., 2020)], and (ii) microglia in the brains of aged mice appear to have β-galactosidase on their cell surface. Note, however, that we do not know whether PMA or LPS induced microglial senescence in our cultures, and much more work would be required to confirm that senescent microglia expose β-galactosidase. If so, this might provide an explanation of why senescent microglia appear to promote neurodegeneration (Bussian et al., 2018; Martínez-Cué and Rueda, 2020).

Aberrant and chronic microglial activation and neuroinflammation are implicated in many neurodegenerative disorders including AD and PD (Perry et al., 2010). In PD patients, GM1 deficiency has been reported, and GM1 replacement may provide some clinical benefit in PD patients (Wu et al., 2012; Schneider et al., 2013). Moreover, β-galactosidase activity is increased in the CSF of PD patients (van Dijk et al., 2013). Thus, it is possible that cell surface or extracellular β-galactosidase degrades protective GM1, resulting in microglial activation and neuronal loss. Furthermore, removal of the terminal galactose residue of GM1, generates GM2 ganglioside, which can be pro-inflammatory (Mizutani et al., 1999). In AD, accumulation of GM2 has been reported (Kracun et al., 1992). Thus, it could be that activated microglia release β-galactosidase that degrades neuroprotective and anti-inflammatory GM1, to produce pro-inflammatory GM2, resulting in chronic inflammation and microglia-mediated neurodegeneration. However, this is a hypothesis that requires testing *in vivo*.

Overall, we have shown that activated microglia can release β-galactosidase, and extracellular β-galactosidase can induce neuronal loss via microglia, such that inhibiting β-galactosidase can prevent inflammatory neuronal loss in culture. Thus, extracellular β-galactosidase is a potential target to prevent neurodegeneration induced by inflammation or aging.

## Data availability statement

The original contributions presented in this study are included in this article/Supplementary material, further inquiries can be directed to the corresponding author.

## Ethics statement

The animal study was approved by the University of Cambridge Animal Welfare and Ethical Review Board. The study was conducted in accordance with the local legislation and institutional requirements.

## Author contributions

EK: Formal analysis, Investigation, Methodology, Visualization, Writing – original draft, Writing – review & editing. JD: Investigation, Writing – review & editing. GB: Conceptualization, Supervision, Writing – original draft, Writing – review & editing.
