# Supplementary figures and images for: Activated microglia release β−galactosidase that promotes inflammatory neurodegeneration
Source: Front Aging Neurosci. 2024 Jan 12;15:1327756. doi: 10.3389/fnagi.2023.1327756 (PMC10811154; doi:10.3389/fnagi.2023.1327756)

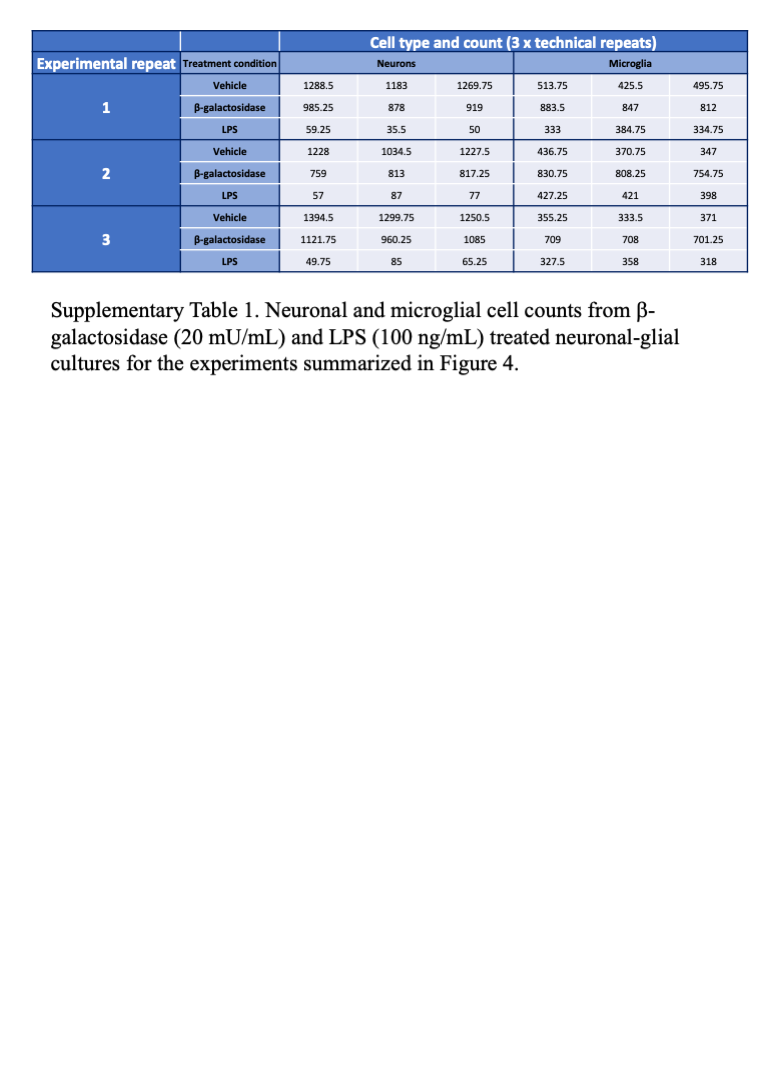

Supplement: Supplementary file 1 [file Image_1.TIFF]

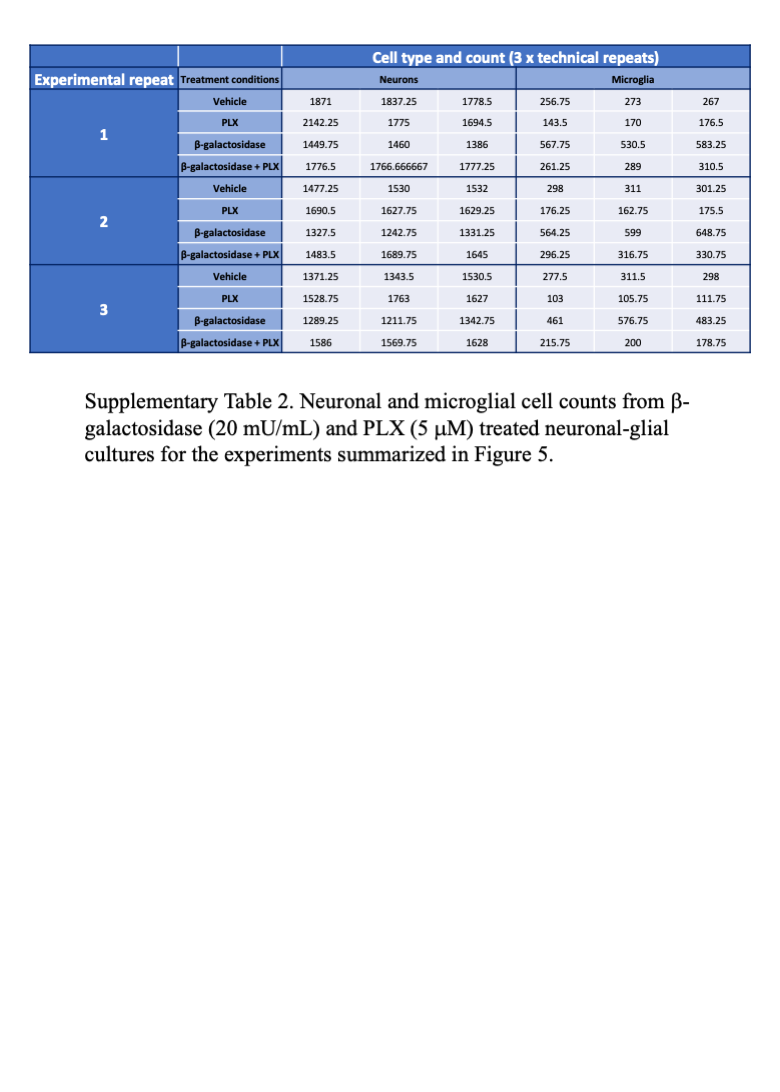

Supplement: Supplementary file 2 [file Image_2.TIFF]

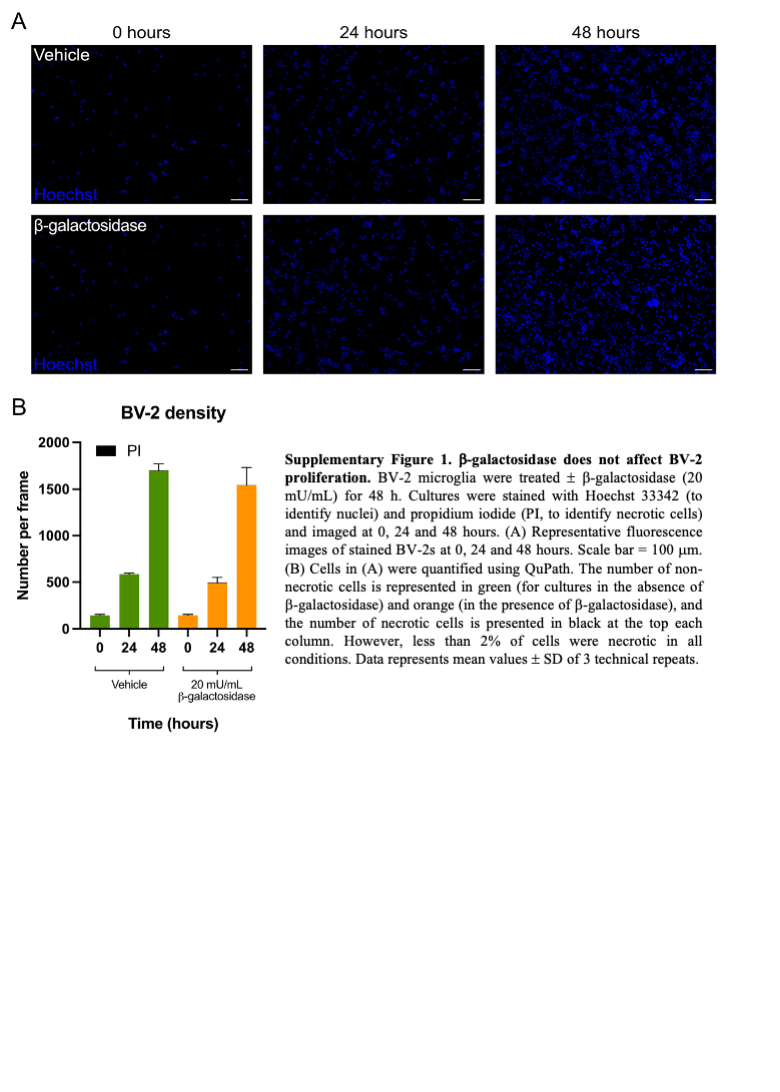

Supplement: Supplementary file 3 [file Image_3.TIFF]

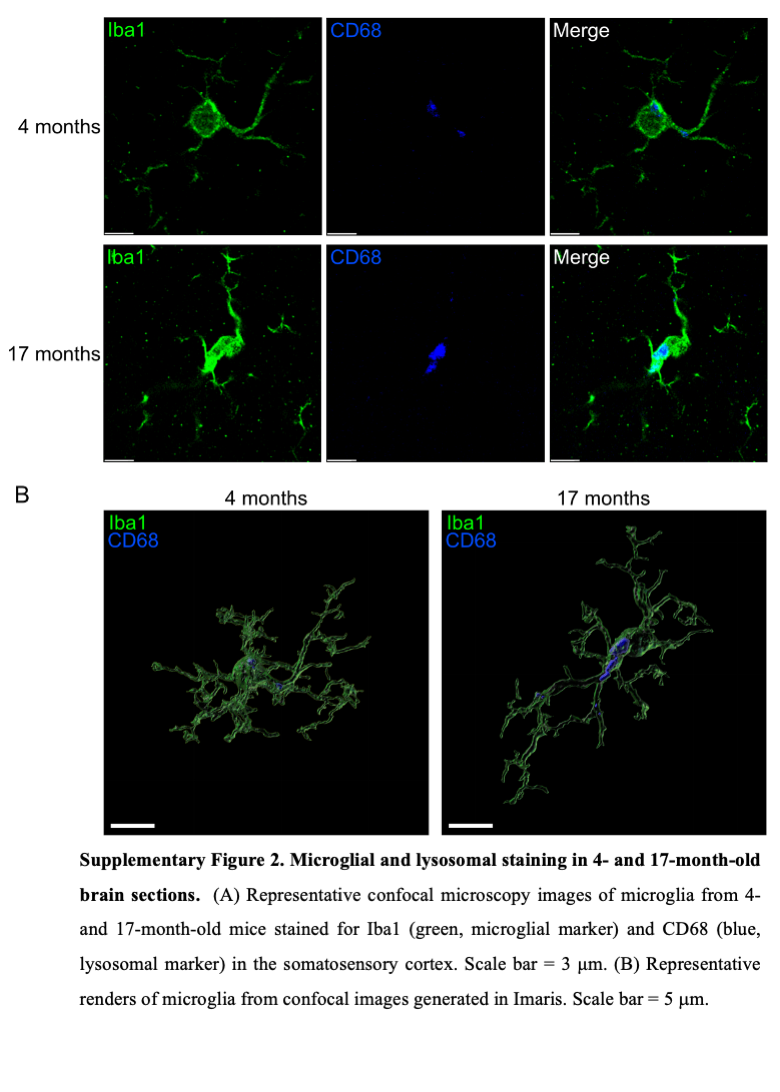

Supplement: Supplementary file 4 [file Image_4.TIFF]

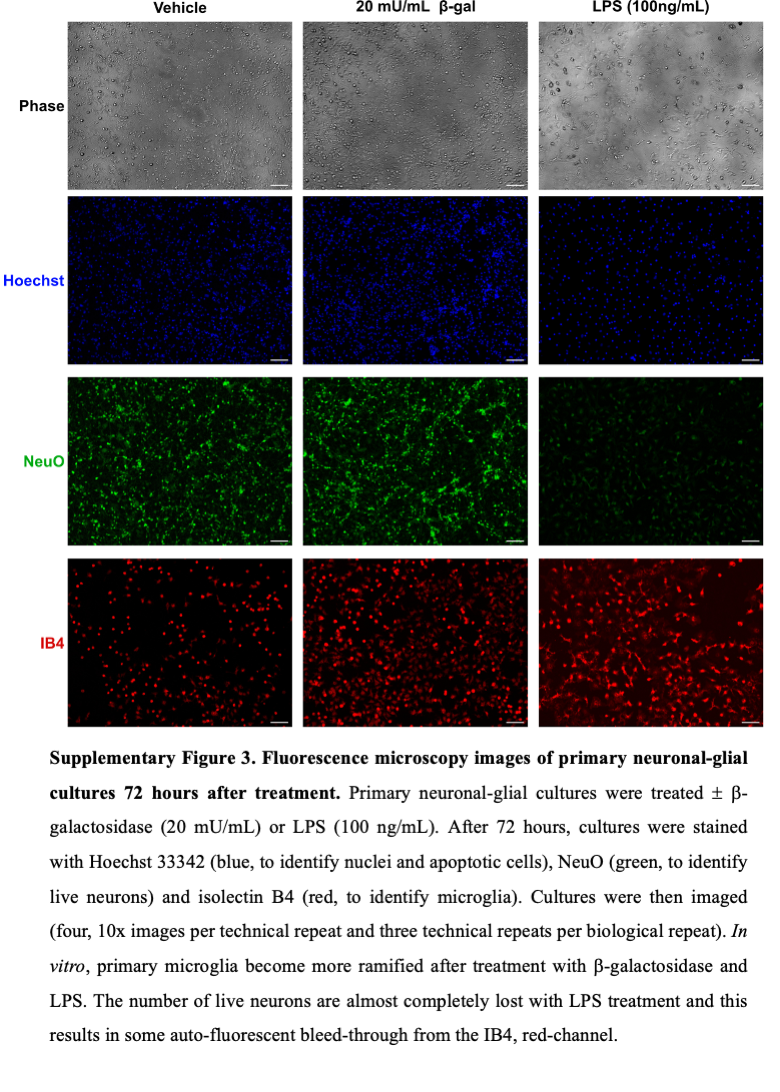

Supplement: Supplementary file 5 [file Image_5.TIFF]

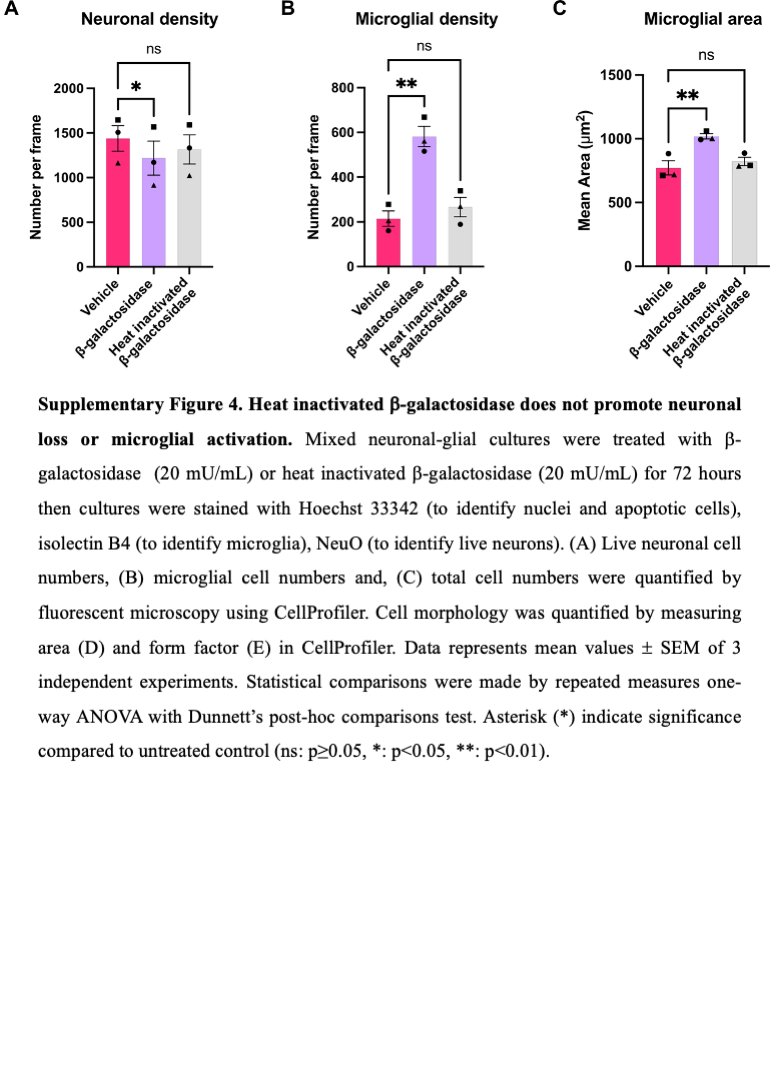

Supplement: Supplementary file 6 [file Image_6.TIFF]

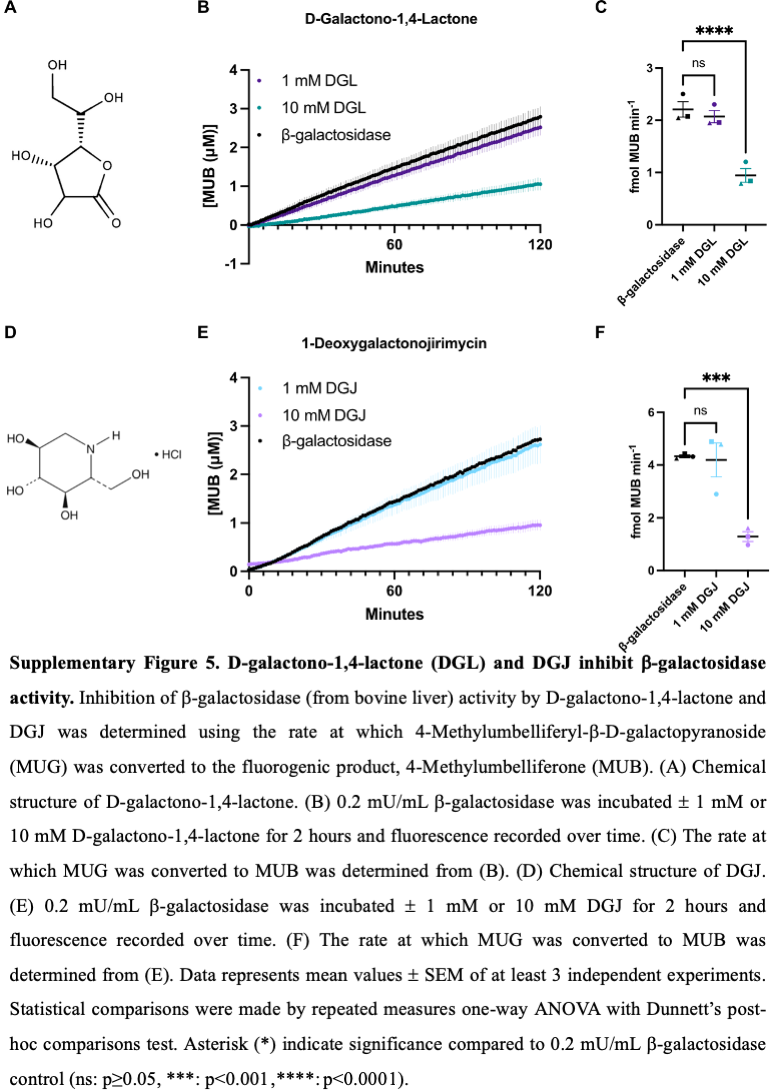

Supplement: Supplementary file 7 [file Image_7.tif]

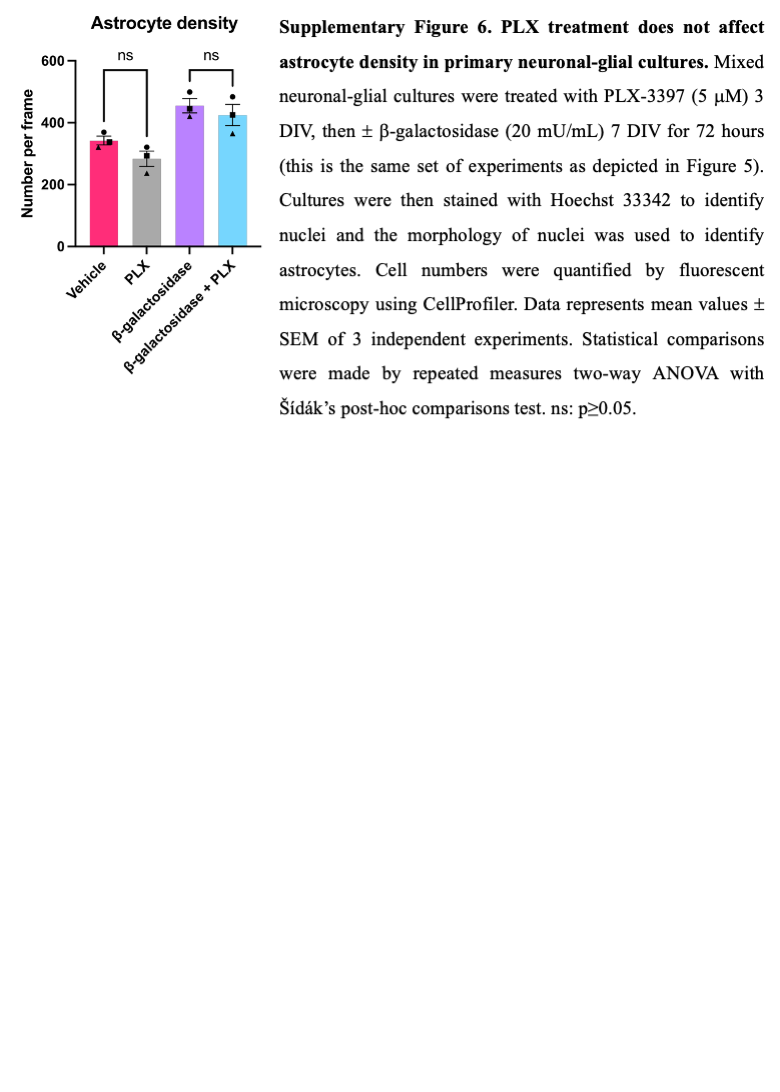

Supplement: Supplementary file 8 [file Image_8.TIFF]
